# A retrospective descriptive study of the characteristics of deliberate self-poisoning patients with single or repeat presentations to an Australian emergency medicine network in a one year period

**DOI:** 10.1186/1471-227X-14-21

**Published:** 2014-08-23

**Authors:** Catherine A Martin, Rose Chapman, Asheq Rahman, Andis Graudins

**Affiliations:** 1 School of Nursing, Midwifery and Paramedicine, Australian Catholic University, Victoria Parade, Fitzroy, Victoria, Australia; 2 Monash Emergency, Dandenong Hospital, Monash Health, David Street, Dandenong, Victoria, Australia; 3 School of Clinical Sciences at Monash Health, Monash University, Clayton, Victoria, Australia; 4 Monash Clinical Toxicology and Addiction Medicine Service, Monash Health, David Street, Dandenong, Victoria, Australia

## Abstract

**Background:**

A proportion of deliberate self-poisoning (DSP) patients present repeatedly to the emergency department (ED). Understanding the characteristics of frequent DSP patients and their presentation is a first step to implementing interventions that are designed to prevent repeated self-poisoning.

**Methods:**

All DSP presentations to three networked Australian ED’s were retrospectively identified from the ED electronic medical record and hospital scanned medical records for 2011. Demographics, types of drugs ingested, emergency department length of stay and disposition for the repeat DSP presenters were extracted and compared to those who presented once with DSP in a one year period. Logistic regression was used to analyse repeat versus single DSP data.

**Results:**

The study determined 755 single presenters and 93 repeat DSP presenters. The repeat presenters contributed to 321 DSP presentations. They were more likely to be unemployed (61.0% versus 39.9%, p = 0.008) and have a psychiatric illness compared to single presenters (36.6% versus 15.5%, p < 0.001). Repeat presenters were less likely to receive a toxicology consultation (11.5% versus 27.3%, p < 0.001) and were more likely to abscond from the ED (7.5% versus 3.4%, p = 0.004). Repeat presenters were more likely to ingest paracetamol and antipsychotics than single presenters. The defined daily dose for the most common antipsychotic ingested, quetiapine, was less in the repeat presenter group (median 1.9 [IQR: 1.3-3.5]) compared with the single presenter group (4 [1.4-9.5]), (OR 0.85, 95% CI 0.74-0.99).

**Conclusion:**

Patients who present repeatedly to the ED with DSP have pre-existing disadvantages, with increased likelihood of being unemployed and having a mental illness. These patients are also more likely to have health service inequities given the greater likelihood to abscond from the ED and lower likelihood of receiving toxicology consultation for their DSP. Early recognition of repeat DSP patients in the ED may facilitate the development of individualised care plans with the aim to reduce repeat episodes of self-poisoning and subsequent risk of successful suicide.

## Background

Deliberate self-poisoning (DSP) is a common cause for frequent presentations to the emergency department (ED), placing an increased burden on the ED [1]. Self-poisoning is the most common method of deliberate self-harm [2]. Presentation with DSP is often perceived negatively by staff involved in the treatment of these patients [3]. Staff attitudes and the patient’s ED experience have been shown to influence the patient’s decision to stay for treatment [4]. A poor ED experience may also exacerbate repeated self-harm behaviour [4]. In addition, an adverse experience may influence whether the patient seeks medical assistance for future self-harm [4]. These findings are significant considering that repeat self-poisoning behaviour results in an increased risk of subsequent successful suicide [5,6].

In an era of increasing demand on the ED [7] and introduction of efficiency initiatives such as the 4-hour rule [8], understanding and describing frequent presenters is an important first step towards development and implementation of strategies to reduce re-attendances. Therefore, the aim of this study was to examine presentations to three Australian ED’s, in a one-year period by repeat DSP status. Characteristics examined were 1) patient characteristics, including age, gender, employment and psychiatric history; 2) presentation characteristics, including length of stay (LOS) data and disposition from the ED; 3) ED interventions with occurrence of psychiatric and toxicological consultations; and 4) type and amount of medications taken in the DSP.

## Methods

This study was a retrospective chart review of DSP presentations to Monash Health’s three emergency departments from January 1^st^ 2011 to December 31^st^ 2011. Ethical approval for the study was obtained from Monash Health Human Research and Ethics Committee and Australian Catholic University. The definition of DSP was based on National Institute for Health and Clinical Excellence (NICE) definition of self-harm where self-harm is defined as ‘any act of self-poisoning or self-injury carried out by an individual irrespective of motivation’ [4]. Repeat poisoning presentations (RPP) were defined as greater than one presentation with DSP in 2011 to any of the three ED’s. A single presentation in 2011, following an episode of DSP, was termed single poisoning presentation (SPP).

A search of the Monash Health’s emergency department electronic database (SYMPHONY Version 2.29, Ascribe plc, Bolton, UK) was conducted to identify DSP presentations. Patients were included if aged ≥ 18 years presenting to Monash Health’s three EDs in 2011 with a presenting complaint of 1) Mental Health involuntary assessment required, 2) Mental Health voluntary requesting treatment or self-harm, 3) overdose/ingestion/poison/toxic exposure were included. The triage notes of each presentation were then screened and those deemed as accidental, recreational and non-DSP presentations were removed. This included presentations requesting psychiatric help or presentations for irrational/disturbed behaviour. Each DSP patient identified from the initial screening then had all their ED presentations in 2011 examined in Scanned Medical Records (SMR) for any missed DSP presentations. Emergency and hospital information about each DSP presentation was obtained from Monash Health SMR. This was then entered into a clinical database specifically designed for toxicology data acquisition. The database included information about the patient’s demographics, previous psychiatric history, toxins taken and amounts, admission and discharge dates, toxicology and psychiatric consults, critical care required, ventilation support required, discharge outcome and follow-up.

Age, employment, and marital status were determined at the index visit. The index visit was defined as the first presentation with DSP to the ED in 2011. The unemployed group included those patients who had identified themselves as unemployed or were on a disability support pension. These items were categorised together as a disability support pension may represent “hidden unemployment” [9]. Employement status distribution was analysed by repeat status. A secondary analysis was also undertaken where patients were categorised into unemployed and ‘other’. ‘Other’ was defined as either being either a student, employed, retired or undertaking home duties. Marital status was categorised into married/de facto relationship or single (single/widowed/separated/divorced).

Previous psychiatric history was obtained from emergency Enhanced Crisis Assessment Treatment Team (ECATT) or psychiatric notes from SMR. A person was deemed to have a previous psychiatric illness if they had documented personality disorder, bipolar affective disorder or schizophrenia.

LOS was determined for 1) patients discharged home from the ED, 2) total hospital LOS for patients admitted to a general ward without requiring critical care stay (time spent in ED and general ward stay) and 3) total hospital stay for patients who required critical care stay (time spent in ED, critical care ward and general ward stay). LOS in the ED was defined as the time spent in ED and short stay unit (observation area of the ED) and was determined as the time recorded on arrival to the ED and discharge time from the ED. Patients staying overnight in ED short-stay/observation unit were considered non-admitted patients**.** LOS for patients admitted to the hospital was determined from SMR as ED arrival time to the time of discharge from the hospital documented in the patients’ medical records. If no discharge time was documented then 1400 hrs was entered into the clinical database. This time was chosen as it was the median discharge time determined for patients discharged from hospital wards with a documented discharge time.

Drugs ingested were categorised into major drug classes. The most common specific agents in each drug class were also examined. Drugs were described by presentation, that is, what percentage of presentations involved ingestion of a particular class of medication. Daily defined dose (DDD) was determined from the Anatomical Therapeutic Chemical/Defined Daily Dose (ATC/DDD) index 2014 World Health Organisation Collaborating Centre for Drug Statistics Methodology website [10]. Comparison of DDD between RPP and SPP was undertaken. A separate analysis of the DDD was also undertaken for patients with multiple frequent DSP presentations to the ED compared with SPP. Multiple frequent DSP attendance was defined as five or more presentations with DSP in 2011 [11]**.**

Summary categorical statistics are expressed as number (percentage). Continuous data were examined for normality using the Shapiro-Wilk and Shapiro-Francia tests. Summary continuous statistics are expressed as mean ± standard deviation if normally distributed or median [inter-quartile range (IQR)] if not. Univariate logistic regression was used to analyse the relationship between all potential predictor variables and the binary outcome variable, RPP versus SPP. The effect size was described as odds ratio (OR) and 95% confidence interval (CI). Statistical significance was considered if the 95% CI included the value one. Hosmer-Lemeshow goodness of fit test was used to assess fit of logistic regression model. Variables were also examined for frequency of missing data. Except for LOS missing data, where single imputation was performed as explained above, complete case analysis was used to handle missing data [12]. Data analysis was performed using Stata version 12 (Stata Corporation, TX, USA).

## Results

### Presentations of DSP

**Figure 1:**
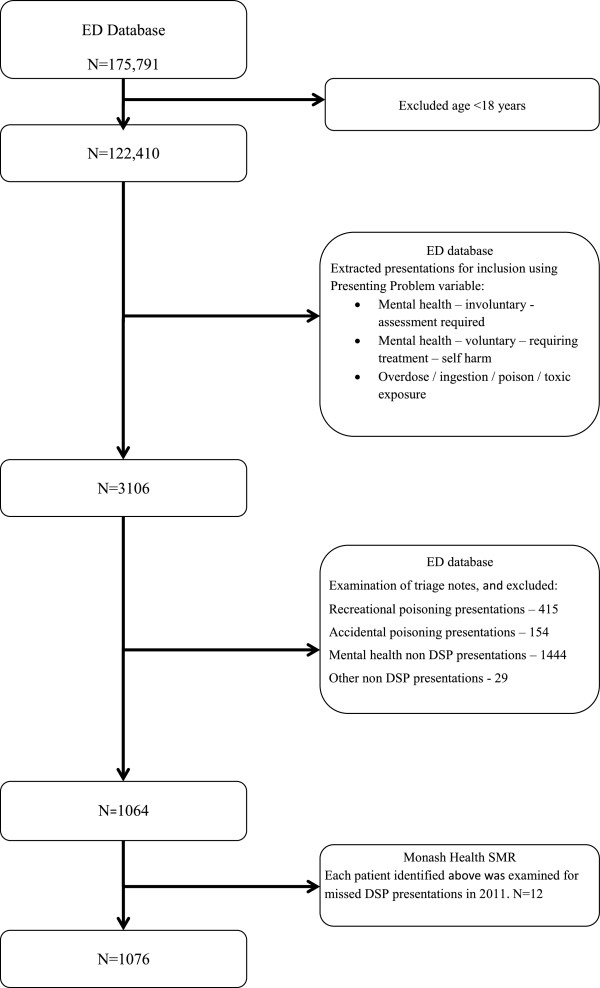
**Flowchart summarizing how DSP presentations to the ED in 2011 were determined.** DSP: deliberate self-poisoning, ED: Emergency Department, SMR: Scanned Medical Records.

A total of 1076 DSP presentations were identified for 2011 (see Figure 1). The majority of these presentations were SPP patient presentations (n = 755, 89.0%) in 2011; whereas 93 RPP patients (11.0%) contributed to 321 presentations (see Table 1). The RPP frequency ranged from 2 to 31 presentations, with a median number of 2 [IQR: 2-3] presentations. Sixty-four patients presented twice; 26 patients presented 3 to 9 times, which contributed to 116 presentations and three patients contributed to 77 presentations in 2011. Forty-two percent of the SPP’s ingested more than one toxin in the DSP, whereas 45% of the RPP group ingested more than one toxin (OR 1.13, 95% CI: 0.65-1.98).

**Table 1:** Characteristics of patients presenting with deliberate self-poisoning in 2011

	**SPP**	**RPP**	**OR**^**¥**^	**95% CI**^**¥**^
Number	755	93		
Age	35.0 [23.7-45.0]	37.0 [26.1-43.6]	0.99	0.98-1.01
Female	485 (64.2)	60 (64.5)	1.10	0.70-1.72
Marital Status				
Married/ Defacto	274 (39.4)	30 (32.6)	1.13	0.47-1.18
Single^€^	422 (60.6)	62 (67.4)		
Employment			1.17^§^	0.97-1.41^§^
Employed	237 (40.7)	22 (28.6)		
Student	50 (8.6)	3 (3.9)		
Unemployed	232 (39.9)	47 (61.0)		
Retired/Pensioner	20 (3.4)	0 (0)		
Home duties	43 (7.4)	5 (6.5)		
Unemployed^δ^	232 (39.9)	47 (61.0)	2.36	1.45-3.85
Psychiatric history^£^	120 (15.5)	34 (36.6)	3.05	1.92-4.85

Data presented as number (%) or median [inter-quartile range]. ^¥^Univariate logistic regression test of SPP versus; ^€^Single includes widows/separated/divorced; ^§^Statistical test of employment distribution; ^δ^Statistical test of unemployed versus ‘others’; ^£^Psychiatric history includes personality disorder, bipolar affective disorder or schizophrenia. *SPP*: Single poisoning presentation, *RPP*: Repeat poisoning presentation.

### Characteristics of DSP patients

No difference was found in the distribution of employment status between the RPP and SPP groups, however when the patients were categorised into unemployed and ‘other’ the RPP patients were more likely to be unemployed than the SPP group (OR 2.36, 95% CI: 1.45-3.85). The RPP patients were more likely to have a previous diagnosis of personality disorder, bipolar affective disorder or schizophrenia than SPP patients (OR 3.05, 95% CI: 1.92-4.85). Although median age was not statistically different between the SPP and RPP groups the RPP group were all younger than 60 years of age (see Table 1).

Table 2 shows that RPP patients were more likely to abscond from the ED and were less likely to have received a Toxicology consultation for their acute poisoning presentation.

**Table 2:** Characteristics of ED disposition, consultations and length of stay

	**SPP**	**RPP**	**OR**^**¥**^	**95% CI**^**¥**^
Number	755	321		
**ED Disposition**				
Home	453 (60.0)	187 (58.3)	0.89	0.64-1.23
ICU/HDU/CCU	54 (7.2)	18 (5.6)	0.77	0.44-1.34
Ventilated	37 (4.9)	8 (2.5)	0.50	0.23-1.08
General ward	86 (11.4)	40 (12.5)	1.11	0.74-1.65
Mental Health	99 (13.1)	38 (11.8)	0.90	0.60-1.34
Involuntary	32 (4.2)	13 (4.1)	0.95	0.49-1.84
Absconded	26 (3.4)	24 (7.5)	2.28	1.28-4.05
Self-discharge	25 (3.3)	12 (3.7)	1.13	0.56-2.28
Other	12 (1.6)	2 (0.6)	NT	
**Consultations**				
Psychiatric consult	457 (60.5)	193 (60.1)	0.98	0.75-1.28
Toxicology consult	205 (27.3)	37 (11.5)	0.35	0.24-0.51
**LOS**				
ED^€^ (hours)	6.5 [4.4-10.8]	7.2 [4.3-12.6]	1.02	0.99-1.06
Non critical care admission^§^ (days)	1.9 [1.3-3.8]	1.8 [1.6-2.8]	0.85	0.70-1.04
Critical care admission^δ^ (days)	2.6 [1.8-3.9]	4.8 [2.5-5.9]	1.15	1.01-1.31

Data presented as number (%) or median [interquartile range]. ^¥^Univariate logistic regression test of SPP versus RPP; ^€^LOS in ED for patients discharged home from ED. ^§^Total hospital LOS, including ED for patients admitted to a general ward; ^δ^Total hospital LOS for patients requiring a critical care admission (CCCU/ICU/HDU), includes ED and ward stay post critical care.. *SPP*: Single poisoning presentation, *RPP*: Repeat poisoning presentation, *ICU*: Intensive care unit, *HDU*: High dependency unit, *CCU*: Coronary care unit, *DSP*: Deliberate self-poisoning, *LOS*: Length of stay, *ED*: Emergency department includes short-stay area, *NT*: Not tested.

### Length of stay

Although not statistically different, the ED LOS for patients who were discharged home from the ED tended to be higher in the RPP group (Table 2). The total hospital stay for patients requiring critical care admission was higher in the RPP group compared with the SPP group (OR 1.15, 95% CI: 1.10-1.31). One patient in the RPP group contributed to three critical care stays.

### Type and amount of medication used in DSP

As a group, benzodiazepines were the most commonly reported toxicant ingested in DSP in 2011 for both the SPP and RPP groups (see Table 3). Diazepam was the most commonly ingested benzodiazepine in both groups but the RPP group had higher DSP presentations associated with diazepam than the SPP group (OR 1.66, 95% CI 1.23-2.25). RPP’s were more likely to ingest paracetamol than SPP patients (OR 1.37, 95% CI: 1.03-1.84) and less likely to take antidepressants (OR 0.64, 95% CI: 0.47-0.88) in overdose. Co-ingestion with alcohol during DSP was high and similar for both groups. Although not statistically different, the RPP patients tended to have a higher number of overdose presentations associated with antipsychotic agents. Quetiapine was the most common antipsychotic ingested for both groups. However, RPP patients more commonly ingested quetiapine for DSP (OR 1.63, 95% CI: 1.06-2.50). Interestingly, the median ingested DDD was lower in the RPP group than for SPP group (median 1.9 [IQR: 1.3-3.5] versus 4 [1.4-9.5], OR 0.85 95% CI: 0.74-0.99) (see Table 4). There was no significant difference in reported median DDD ingested between the two groups for any of the other classes of medications.

**Table 3:** Comparison of drugs ingested by class for single and repeat presenters

**Drug Class**	**Most common specific agents in class**	**SPP**^**€**^**presentations (N = 752)**	**RPP**^**€**^**presentations (N = 315)**	**OR**^**¥**^	**95% CI**^**¥**^
Benzodiazepines		296 (39.4)	135 (42.9)	1.12	0.85-1.46
	Diazepam	146 (19.4)	90 (28.6)	1.66	1.23-2.25^*^
	Temazepam	74 (9.8)	24 (7.6)	0.76	0.47-1.22
	Alprazolam	64 (8.5)	21 (6.7)	0.76	0.46-1.28
Alcohol (Beverage)		245 (32.5)	95 (30.0)	0.89	0.67-1.19
Paracetamol		179 (23.8)	96 (30.5)	1.37	1.03-1.84^*^
Antidepressants		217 (28.9)	65 (20.6)	0.64	0.47-0.88^*^
SSRI^£^		89 (11.8)	20 (6.3)	0.51	0.31-0.84^*^
	Escitalopram/Sertraline	51 (6.8)	15 (4.8)	0.69	0.38-1.24
SNRI	Venlafaxine/Desvenlafaxine/Duloxetine^§^	47 (6.3)	19 (6.0)	0.96	0.56-1.67
TCA		39 (5.2)	7 (2.2)	NT	
Antipsychotics		99 (13.2)	55 (17.5)	1.40	0.97-2.00
	Quetiapine	60 (8.0)	39 (12.4)	1.63	1.06-2.50^*^
	Olanzapine	11 (1.5)	7 (2.2)	NT	
NSAIDs		81 (10.8)	30 (9.5)	0.87	0.56-1.36
Other drugs		266 (35.4)	100 (31.8)	0.85	0.64-1.12
	Opioids	61 (8.1)	11 (3.5)	0.41	0.21-0.79^*^
	Zolpidem/Zopiclone	32 (4.3)	13 (4.1)	0.97	0.50-1.87

Data presented as number (%). ^¥^Univariate logistic regression test of SPP versus RPP, ^€^Data missing: three for single presenters and 6 for repeat presenters, information either not in scanned medical records or described as unknown, ^*^Significant difference, ^£^Data presented for all SSRI and the three most common ingested, ^§^Only these SNRI’s ingested in DSP. *SPP*: Single poisoning presentation, *RPP*: Repeat poisoning presentation, *OR*: Odds ratio, *CI*: Confidence interval, *SSRI*: Serotonin-specific reuptake inhibitor, *SNRI*: Serotonin–norepinephrine reuptake inhibitor, *TCA*: Tricyclic antidepressants, *NSAID*: Non-steroidal anti-inflammatory, *NT*: Not tested. Note: frequencies add up to greater than 100% as DSP may be associated with more than one toxin.

**Table 4:** Comparison of amount of drug ingested for single and repeat deliberate self-poisoning presenters defined by daily dose of drug (DDD)

	**N**	**SPP**	**N**	**RPP**	**OR**^**¥**^	**95% CI**^**¥**^
Benzodiazepine	217	6 [4-14]	101	10 [5-16]	1.01	1.00-1.02
Diazepam	103	6 [3-15]	68	10 [4.4-15]	1.02	0.99-1.05
Temazepam	67	5.5 [3-10]	20	5.5 [2.8-12.3]	0.99	0.91-1.08
Alprazolam	35	10 [5-20]	13	24 [20-32]	1.01	1.00-1.03
Paracetamol	162	2.2 [1.3-4.4]	85	3.3 [1.7-5.0]	1.06	0.98-1.15
Antidepressant	147	15 [6-28]	42	20 [10-32]	1.01	0.99-1.02
SSRI	100	15 [7-28]	25	20 [10-25]	1.01	0.99-1.02
Antipsychotic	61	4.5 [1.8-10.5]	39	3 [1.3-5]	0.98	0.93-1.03
Quetiapine	40	4 [1.4-9.5]	26	1.9 [1.3-3.5]	0.85	0.74-0.99^*^
Anticonvulsant	25	4.5 [1.3-13.3]	12	7.3 [3.3-12.8]	1.00	0.92-1.09

Data presented as median [interquartile range]. ^¥^Univariate logistic regression test of SPP versus RPP, *Statistically significant. *N*: Number, *SPP*: Single poisoning presentation, *RPP*: Repeat poisoning presentation, *OR*: Odds ratio, *CI*: Confidence interval.

### Frequent DSP attenders

A separate analysis for those with five or more DSP presentations in 2011 showed that these patients took more DDDs of Paracetamol compared to SPP patients (median 3.8 [IQR: 2.6-6] versus 2.2 [IQR: 1.3-4.4]; OR 1.14, 95% CI: 1.04-1.25).

### Inpatient mortality from DSP

There were no inpatient deaths as a result of DSP during the study period.

### Missing data

The majority of variables examined had none or few missing data points as data is collected for key performance indicators for the ED. Variables with the most missing data included marital status (6.8%), employment status (19.6%), toxicology consultation (12.9%) and psychiatric consultations (23.2%). For the variables marital status and employment, the majority of missing data occurred in the SPP group.

## Discussion

This study confirms the results of a number of previous studies examining DSP repeat presenters observing that, this patient group was more likely to be unemployed and have a mental health illness compared to those presenting only once [13,14]. This study found that RPP patients were more likely to be disadvantaged in other ways such as, they were more likely to abscond before care was completed and less likely to have a toxicology assessment even though similar doses of drug were ingested for most classes of medication. A similar severity of poisoning in both patient groups is suggested by the fact that ingested drug dose for most toxins was similar and the frequency of ICU admission was also not significantly different.

The proportion of DSP patients with recurrent presentations in the same year in this study was similar to that reported in other studies [14-17]. However, two studies reported higher re-presentation proportions [13,18]. One of these reported that 21% of DSP patients had recurrent presentations; however this was a retrospective study with a study period of ten years, which may have influenced the result [13]. The other study reported that 18% of DSP patients re-presented. The difference in representation proportion may be due to the prospective nature of the study as DSP patients, who met the study inclusion criteria, were consented to be followed over time [18].

Similar to our study, previous studies have also found that RPP patients were more likely to be unemployed [13,18,19], single [14], live alone [20], or have a psychiatric history [13,18,19]; particularly personality disorder or schizophrenia [19] than SPP patients.

Gender and age differences in RPP patients have been less consistent in previous studies, with two studies finding no gender influence on RPP behaviour [15,16]. However, two other studies found the opposite, with female gender being a significant factor in RPP behaviour [13,14]. Additionally, we found no association with age and RPP status. This is consistent with another Australian study [14]. Other studies have noted that RPP patients tended to be older than SPP patients [15,19]. It is possible that there are geographical and cultural differences between studies. For example, the catchment area of the present study included a large proportion of people born overseas [21]. However, it was beyond the scope of this study to examine this further.

This study also found no difference between SPP and RPP patients in their disposition from the ED. However, an older study of self-poisoning found that RPP patients were less likely to be admitted to a hospital ward than SPP patients [15]. As RPP patients had lower triage category scores than SPP patients the authors hypothesised that RPP patients were less sick and hence less likely to be admitted.

Differences in length of stay between SPP and RPP groups have not been well characterised. A study undertaken in 1999 examined hospital LOS of the index visit for the RPP group versus the SPP group. It was found that the RPP group had statistically significant longer hospital LOS than the SPP group, but the authors felt the difference was not clinically significant [14]. Our study found that RPPs had a significantly longer LOS if critical care admission was required. However, the number of critical care admissions in the RPP group was not large and one patient contributed to three critical care admissions. In older studies of DSP patients, the proportion of patients admitted to hospital was considerably greater than in this study and other more contemporary studies. A study from 20 years ago reported that 70% of all DSP patients were admitted to an in-patient bed [16], which was considerably higher than in the present study. The lower in-patient admission rate may reflect the current practice of utilising ED short stay or observation units for patients with projected short lengths of stay (<48 hours) as is often the case with DSP patients.

Very few studies have examined whether the characteristics of toxicants ingested by SPP or RPP patients vary. In a retrospective study that examined the characteristics of patients presenting with DSP to a South Korean ED over a nine year period; the authors found that RPP patients were more likely to ingest sedatives or antidepressants whereas SPP patients tended to use sedatives or analgesics [13]. In an Australian study, the RPP characteristics for patients presenting to a Victorian hospital over a two-year period between 1993 and 1994 were examined. Those with greater than one presentation with DSP tended to take a single drug in the overdose compared to those that presented once [15]; this was not the case in this present study. However, similar to the current study, paracetamol overdose was higher in the RPP group. In another Australian study the class of drug ingested at the index visit in SPP and RPP patients was examined within one year period [14]. Benzodiazepines were most commonly ingested followed by paracetamol in both groups. In the RPP group, the third most common drug class was antipsychotics, whereas for the SPP group it was antidepressants [14]. This observation parallels the results of our study. Our study shows that RPP had higher use of quetiapine associated DSP but the ingested dose was lower in this group. Repeat DSP is often associated with pre-existing mental health conditions, such as schizophrenia and bipolar affective disorder [19], which allows easier access to antipsychotic prescription medication such as quetiapine. Concerns about the trend of increased rates of quetiapine prescribing and ambulance call outs associated with quetiapine use in recent years have been raised in Australia [22]. This study also reveals that although there was no significant difference in overall benzodiazepine ingestion between the two groups, the RPP group had statistically higher use of diazepam. This may reflect greater access to this drug in recurrent presenters.

Although there are methodological differences and over a decade has lapsed between this current study and the two previous Australian studies, they all show that the top four classes of drugs used in DSP, excluding alcohol, are benzodiazepine, paracetamol, antidepressants and antipsychotics [14,15]. The impulsivity associated with DSP [23] may explain why paracetamol was the second most common ingested medication for patients who had repeated presentations following DSP in this current study.

This study confirms the results of other studies that alcohol co-ingestion is common in patients with DSP [1,23]. This current study found no difference in frequency of alcohol co-ingestion between the two groups, however, we did not examine chronic alcohol use as a predictor for RPP.

We recently reported that ED nursing and medical staff felt empathy towards DSP patients who presented once but frustration towards DSP patients who presented repeatedly [24,25]. The association of psychosocial history, unemployment and psychiatric illness, and more frequent absconding behaviour from the ED may result in staff feeling that they are dealing with patients who are difficult to manage, do not take responsibility for their actions, and do not want help. This finding is especially significant for those patients who re-present on numerous occasions. Many of the RPP patients were considered to be attention seeking, taking up resources and some staff queried whether the ED was the right place for these patients [24,25]. The sub-analysis of the most frequent DSP attenders suggested that the severity of poisoning was similar to the single presenters. For those that ingested paracetamol the ingested dose was greater in the frequent repeat presenters, with a median DDD of 3.8. This is within the range of expected toxic ingested dose requiring antidote treatment [26]. This suggests risk assessment in the ED is important in this group.

The ED has an important role in the recognition of patients at risk of recurrent self-poisoning and re-presentation to hospital. Detection of patients at risk of re-harming may allow the implementation of evidence based interventions designed to reduce this phenomenon. This may include measures such as frequent postcards sent to DSP patients, telephone follow up, or follow up in self-harm clinics once discharged [27]. An increase in the severity of repeat DSP is associated with increased risk of future suicide completion [28]. As a result, a database of DSP patient presentations could identify those in the RPP group. Coupled with regular multidisciplinary clinical meetings, this would facilitate the development and implementation of specific care plans for patients.

The retrospective nature of this study meant that we were reliant on the integrity of the medical record data. The variables marital and employment status, documentation of toxicology and psychiatric consult, all had high levels of missing data, which may have biased the results. Variables regarding the type and amount of medications used in DSP episodes were not collected for some patients, such as those with altered conscious state, which may also have influenced the results. We used the ED presenting diagnosis to determine self-poisoning presentations and may have missed some patients presenting with DSP. Also, we only examined repeat presentations in a one year period and some patients in the single presentation group may have re-presented beyond the boundaries of the study time-frame. Patients may have presented to other hospital networks during the study period, which means that patients identified as single presentations may actually be recurrent presenters. While there were no in-hospital deaths in this cohort, we did not analyse coroner’s data to assess for completed suicide following DSP in the community. Variables that may have helped to explain repeater behaviour were not collected for this study. These included previous DSP episodes, drug and alcohol history, and associated self-injury with the DSP. Therefore, further empirical studies investigating those patients who present to the ED following repeated DSP presentations are required.

## Conclusion

Our study provides more contemporary information regarding the characteristics of patients who present with repeated self-poisoning in an Australian setting. This patient group has pre-existing disadvantages, with increased likelihood of being unemployed and having a pre-existing mental health problem. They are also more likely to have health service inequities given the greater likelihood to abscond from the ED and lower likelihood of receiving toxicology consultation for their poisoning. However, repeat presenters had a similar severity of DSP as determined by frequency of critical care admission and similar doses and classes of drug ingested compared to single presenters. ED staff are well placed to facilitate the development and implementation of evidence based intervention and specific care plans for these patients.

## Competing interests

The authors declare that they have no competing interests.

## Authors' contributions

CM carried out data input, statistical analysis and interpretation of data; and drafting and revising manuscript. RC conceived the study and participated in data interpretation, drafting and revising manuscript. AG carried out data input, and participated in data interpretation, drafting and revising manuscript. AR carried out drafting and revising manuscript. All authors read and approved the final manuscript.

## Pre-publication history

The pre-publication history for this paper can be accessed here:

http://www.biomedcentral.com/1471-227X/14/21/prepub

## References

[B1] Hendrix L Verelst S Desruelles D Gillet JB Deliberate self-poisoning: characteristics of patients and impact on the emergency department of a large university hospital EMJ 2013 30 1 e9 10.1136/emermed-2011-201033 22328636

[B2] Gunnell D Bennewith O Peters TJ House A Hawton K The epidemiology and management of self-harm amongst adults in England J Public Health 2005 27 67 73 10.1093/pubmed/fdh192 15564277

[B3] Saunders KE Hawton K Fortune S Farrell S Attitudes and knowledge of clinical staff regarding people who self-harm: a systematic review J Affect Disord 2012 139 205 216 10.1016/j.jad.2011.08.024 21925740

[B4] National Institute for Clinical Excellence Self-harm: The short-term physical and psychological management and secondary prevention of self-harm in primary and secondary care National Clinical Practice Guideline Number 16 2004 Level 1A, City Tower, Piccadilly Plaza, Manchester M1 4BT: National Institute for Health and Clinical Excellence

[B5] Crandall C Fullerton-Gleason L Aguero R LaValley J Subsequent suicide mortality among emergency department patients seen for suicidal behavior Acad Emerg Med 2006 13 435 442 10.1111/j.1553-2712.2006.tb00322.x 16531601

[B6] Suominen K Isometsa E Suokas J Haukka J Achte K Lonnqvist J Completed suicide after a suicide attempt: a 37-year follow-up study Am J Psychiatr 2004 161 562 563 10.1176/appi.ajp.161.3.562 14992984

[B7] Lowthian JA Curtis AJ Jolley DJ Stoelwinder JU McNeil JJ Cameron PA Demand at the emergency department front door: 10-year trends in presentations Med J Aust 2012 196 128 132 10.5694/mja11.10955 22304608

[B8] Mason S Weber EJ Coster J Freeman J Locker T Time patients spend in the emergency department: England’s 4-hour rule-a case of hitting the target but missing the point? Ann Emerg Med 2012 59 341 349 10.1016/j.annemergmed.2011.08.017 22088495

[B9] Cai L Gregory B Unemployment duration and inflows onto the disability support pension program: evidence from FaCS LDS data Aust Econ Rev 2005 38 233 252 10.1111/j.1467-8462.2005.00371.x

[B10] Anatomical Therapeutic Chemical/ Defined Daily Dose (ATC/DDD) index 2014 World Health Organisation Collaborating Centre for Drug Statistics Methodology http://www.whocc.no/atc_ddd_index/

[B11] Locker TE Baston S Mason SM Nicholl J Defining frequent use of an urban emergency department Emerg Med J 2007 24 398 401 10.1136/emj.2006.043844 17513534 PMC2658272

[B12] Groenwold RH Donders AR Roes KC Harrell FE Jr Moons KG Dealing with missing outcome data in randomized trials and observational studies Am J Epidemiol 2012 175 210 217 10.1093/aje/kwr302 22262640

[B13] Oh SH Park KN Jeong SH Kim HJ Lee CC Deliberate self-poisoning: factors associated with recurrent self-poisoning Am J Emerg Med 2011 29 908 912 10.1016/j.ajem.2011.03.015 21641159

[B14] Carter GL Whyte IM Ball K Carter NT Dawson AH Carr VJ Fryer J Repetition of deliberate self-poisoning in an Australian hospital-treated population Med J Aust 1999 170 307 311 10327971 10.5694/j.1326-5377.1999.tb127783.x

[B15] Taylor DM Cameron PA Eddey D Recurrent overdose: patient characteristics, habits, and outcomes J Accid Emerg Med 1998 15 257 261 10.1136/emj.15.4.257 9681311 PMC1343140

[B16] Boyes AP Repetition of overdose: a retrospective 5-year study J Adv Nurs 1994 20 462 468 10.1111/j.1365-2648.1994.tb02382.x 7963051

[B17] Prescott K Stratton R Freyer A Hall I Le Jeune I Detailed analyses of self-poisoning episodes presenting to a large regional teaching hospital in the UK Br J Clin Pharmacol 2009 68 260 268 10.1111/j.1365-2125.2009.03458.x 19694747 PMC2767291

[B18] Riedi G Mathur A Seguin M Bousquet B Czapla P Charpentier S Genestal M Cailhol L Birmes P Alcohol and repeated deliberate self-harm: preliminary results of the French cohort study of risk for repeated incomplete suicides Crisis 2012 33 358 363 22759664 10.1027/0227-5910/a000148

[B19] Morton MJ Prediction of repetition of parasuicide: with special reference to unemployment Int J Soc Psychiatry 1993 39 87 99 10.1177/002076409303900202 8340216

[B20] James IP Self-poisoning and alcohol Lancet 1972 2 1260 1261 4117758 10.1016/s0140-6736(72)92322-7

[B21] Australian Bureau of Statistics http://www.censusdata.abs.gov.au/census_services/getproduct/census/2011/quickstat/0

[B22] Heilbronn C Lloyd B McElwee P Eade A Lubman DI Trends in quetiapine use and non-fatal quetiapine-related ambulance attendances Drug Alcohol Rev 2013 32 405 411 10.1111/dar.12028 23350582

[B23] Dir AL Karyadi K Cyders MA The uniqueness of negative urgency as a common risk factor for self-harm behaviors, alcohol consumption, and eating problems Addict Behav 2013 38 2158 2162 10.1016/j.addbeh.2013.01.025 23454879

[B24] Chapman R Martin C Perceptions of Australian emergency staff towards patients presenting with deliberate self-poisoning: a qualitative perspective Int Emerg Nurs 2014 doi: 10.1016/j.ienj.2014.03.002 10.1016/j.ienj.2014.03.002 24768529

[B25] Martin C Chapman R A mixed method study to determine the attitude of Australian emergency health professionals towards patients who present with deliberate self-poisoning Int Emerg Nurs 2014 22 98 104 10.1016/j.ienj.2013.09.002 24207085

[B26] Daly FFS Fountain JS Murray L Graudins A Buckley NA Guidelines for the management of paracetamol poisoning in Australia and New Zealand – explanation and elaboration MJA 2008 188 296 301 18312195 10.5694/j.1326-5377.2008.tb01625.x

[B27] Carter GL Clover K Whyte IM Dawson AH D’Este C Postcards from the EDge: 5-year outcomes of a randomised controlled trial for hospital-treated self-poisoning Br J Psychiatry 2013 202 372 380 10.1192/bjp.bp.112.112664 23520223

[B28] Carter G Reith DM Whyte IM McPherson M Repeated self-poisoning: increasing severity of self-harm as a predictor of subsequent suicide Br J Psychiatry 2005 186 253 257 10.1192/bjp.186.3.253 15738507

